# KeepRunning: A MoCap-Based Rapid Test to Prevent Musculoskeletal Running Injuries

**DOI:** 10.3390/s23239336

**Published:** 2023-11-22

**Authors:** Javier Rodríguez, Javier Marín, Ana C. Royo, Luis Padrón, Manuel Pérez-Soto, José J. Marín

**Affiliations:** 1 IDERGO (Research and Development in Ergonomics), I3A (Instituto de Investigación en Ingeniería de Aragón), University of Zaragoza, C/María de Luna, 3, 50018 Zaragoza, Spaincrisroyo@unizar.es (A.C.R.); lpadron@unizar.es (L.P.); manuel.perez@unizar.es (M.P.-S.); jjmarin@unizar.es (J.J.M.); 2 Department of Design and Manufacturing Engineering, University of Zaragoza, C/María de Luna, 3, 50018 Zaragoza, Spain; 3 Department of Biomedical Engineering, University of Zaragoza, C/Mariano Esquillor s/n, 50018 Zaragoza, Spain

**Keywords:** injury prevention, biomechanics, running pattern, optical motion capture (MoCap), reproducibility, running technique, minimal detectable change (MDC)

## Abstract

The worldwide popularisation of running as a sport and recreational practice has led to a high rate of musculoskeletal injuries, usually caused by a lack of knowledge about the most suitable running technique for each runner. This running technique is determined by a runner’s anthropometric body characteristics, dexterity and skill. Therefore, this study aims to develop a motion capture-based running analysis test on a treadmill called KeepRunning to obtain running patterns rapidly, which will aid coaches and clinicians in assessing changes in running technique considering changes in the study variables. Therefore, a review and proposal of the most representative events and variables of analysis in running was conducted to develop the KeepRunning test. Likewise, the minimal detectable change (MDC) in these variables was obtained using test–retest reliability to demonstrate the reproducibility and viability of the test, as well as the use of MDC as a threshold for future assessments. The test–retest consisted of 32 healthy volunteer athletes with a running training routine of at least 15 km per week repeating the test twice. In each test, clusters of markers were placed on the runners’ body segments using elastic bands and the volunteers’ movements were captured while running on a treadmill. In this study, reproducibility was defined by the intraclass correlation coefficient (ICC) and MDC, obtaining a mean value of ICC = 0.94 ± 0.05 for all variables and MDC = 2.73 ± 1.16° for the angular kinematic variables. The results obtained in the test–retest reveal that the reproducibility of the test was similar or better than that found in the literature. KeepRunning is a running analysis test that provides data from the involved body segments rapidly and easily interpretable. This data allows clinicians and coaches to objectively provide indications for runners to improve their running technique and avoid possible injury. The proposed test can be used in the future with inertial motion capture and other wearable technologies.

## 1. Introduction

Today, running has become one of the most popular and widely practised sports due to its health benefits, low cost and easy accessibility [1]. However, the popularisation of this sport has resulted in a high rate of musculoskeletal injuries to the lower extremities [2]. The top five pathologies with the highest incidence are: Achilles tendinopathy (9.1–10.9%), medial tibial stress syndrome (13.6–20.0%), patellofemoral pain syndrome (6.3%), plantar fasciitis (4.5–10.0%) and iliotibial band syndrome (7.9%) [1,3,4,5,6]. Numerous studies have reported that the incidence of running-related musculoskeletal injuries (RRMIs) is between 20% and 79% of the running population [7,8,9,10].

Adequate identification of biomechanical running patterns (i.e., the set of parameters that characterise an athlete’s running) by health professionals and coaches is essential for the prevention of RRMIs [3,4]. Biomechanical analysis of runners allows finding the best subset of running characteristics. This analysis is useful to better understand the possible relationship between biomechanical variables and injuries, allowing the identification of relevant differences in sports and clinical gait patterns [11,12]. Therefore, having objective data on biomechanical running patterns provides a basis for the investigation of risk factors associated with RRMIs [3,4], allowing better diagnostic and training decisions to be made.

However, this pattern identification is difficult to achieve because the pattern of each runner is unique, and there is no biomechanically perfect and generalisable running style [1]. Therefore, it is necessary to understand the dexterity, and the specific biomechanical and anthropometric body characteristics of each runner to design and prescribe the most appropriate technique in a personalised and individualised manner to avoid future RRMIs.

Different technologies have been used to obtain running patterns according to the literature. The most common are inertial sensors, instrumented treadmills and optical sensors [13,14,15,16,17,18,19]. The major differences found between them are that inertial sensors are less accurate than optical sensors, but they can be used outdoors [20], while instrumented treadmills only allow the study of spatio-temporal variables and ground reaction forces [21].

Motion capture (MoCap) systems based on optical technology can be used to conduct a detailed biomechanical analysis of running by obtaining three-dimensional (3D) human movement objectively from reflective markers [22]. Optical systems are highly accurate and stable and are therefore considered the gold standard [1,7,13,22,23]. These systems define the 3D marker trajectory, displacement, angular and linear velocity and limb acceleration, which are helpful data in running analyses [22]. For this large dataset to be interpreted by trainers and clinicians, it is possible to obtain objective variables that are characteristic of the running technique and must be presented in a clear and comprehensive format [24].

These variables are defined as numerical magnitudes that allow interpreting results and drawing clinical conclusions (i.e., indicators that characterise the running pattern) [25]. Nevertheless, the number of possible variables to select in a study is quite high because each body segment to be studied presents three movement curves in the three anatomical planes (sagittal, frontal and transversal). In addition, variables can be calculated as point data at any instant on the curve or as ranges of movement between two points.

Along these lines, each study uses different specific variables for running analysis [26,27,28,29,30,31,32], and no common or transversal parameters have been found to be taken as a general reference (i.e., the variables selected depend on the objective of each study). Therefore, variables must be as standardised, representative and intuitive as possible to assist clinicians and trainers in the prevention of runner injuries.

Regarding the variables related to RRMIs, the most common injuries develop gradually over time and are thought to be associated with a complex and multifactorial aetiology [1,33,34]. These factors are divided into intrinsic (personal or internal) and extrinsic (environmental or external) factors [35,36], which can interact with each other along with the stresses applied to body tissues during running. The combination of these factors predisposes runners to develop an RRMI [7,35]. Intrinsic factors include biomechanical factors, as described by Moore [36], which can be most influenced by coaches and health professionals.

In addition, the use of these systems in daily clinical practice requires simplicity to set up and intuitiveness to achieve rapid measurements [37]. Therefore, a new rapid and simple test must be developed based on MoCap to interpret the large amount of data obtained because these tests are usually quite time-consuming, and the interpretation of the results is complex. This test should facilitate individualised running analysis because it reduces operating time, simplifies data collection and facilitates the interpretation of results.

However, a MoCap-based test is conditioned by measurement errors or inaccuracies due to the intrinsic variations in running, soft-tissue movements, relative movements between clusters of markers and the skin, positioning, instrument accuracy, running event detection and anatomical calibration [20]. MoCap remains excellent for validating the test due to its high accuracy and versatility, although it is conditioned by these factors.

Thus, the quality and validity of the test must be assessed by applying indicators or metrics. Among the existing accuracy indicators, reproducibility is the most general and critical indicator to be considered in a MoCap analysis system [38]. Satisfactory reproducibility results indicate that, for the same conditions, the system produces similar data each time it is used, indicating that the system is sufficiently accurate to compare a subject’s results over time. In this regard, despite the widespread use of 3D motion analysis in laboratories, only relatively few studies have evaluated the reproducibility of running kinematics using this type of test [13,14,15,16,17,18,19].

The test should enable detecting whether the biomechanical pattern has changed between one assessment and the next after the indications or corrections proposed by the specialists. Whether the changes are real or produced by system errors must be determined. According to Nüesch et al. [15], whether the change between two sessions is simply due to measurement errors and, therefore, not attributable to real changes in the runner can be identified using the reproducibility index through the minimal detectable change (MDC). This index allows judging the probability of a real improvement (or deterioration) in a subject [20] because it represents the degree of a representative change of a real change and is expressed in the same unit of measurement as the measurement itself (degrees of movement or dimensional values of displacement) [16]. There is a need for studies that show and discuss the practical application of the results obtained from MDC in running analysis.

Therefore, the main objective of this paper is to present a running analysis test based on the optical MoCap and the treadmill called KeepRunning, which allows individualised monitoring of runners and is aimed at injury prevention. For this purpose, the following actions were accomplished:

(i) Define the running events;
(ii) Select the most relevant variables with the greatest influence on the prevention of RRMIs considering the literature and the authors’ criteria, according to the convenience of the KeepRunning test;
(iii) Finally, assess the reproducibility of the running test by conducting a test–retest reliability evaluation on healthy athletes. Test–retest consists of repeating the test twice.

This approach is expected to obtain an objective, reproducible and interpretable test that allows the evaluation and comparison of the results between two captures and quantifies the changes in the running pattern after the coach’s indications. Therefore, this new test can provide a tool where the change of each variable is reflected. Thus, a specialist can evaluate whether positive or negative changes are produced in the athlete’s running technique (i.e., the set of movements and gestures involved in running) based on the easy interpretation of the results and his or her knowledge. This allows the specialist to detect inappropriate running patterns that could lead to RRMI and provide recommendations for improving technique.

## 2. Materials and Methods

### 2.1. KeepRunning Analysis Test

#### 2.1.1. Sensor Technology

**Figure 1:**
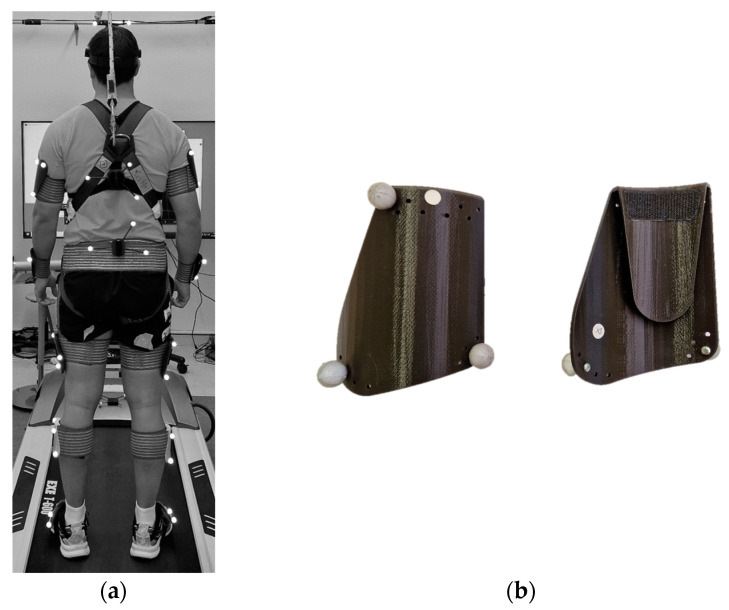

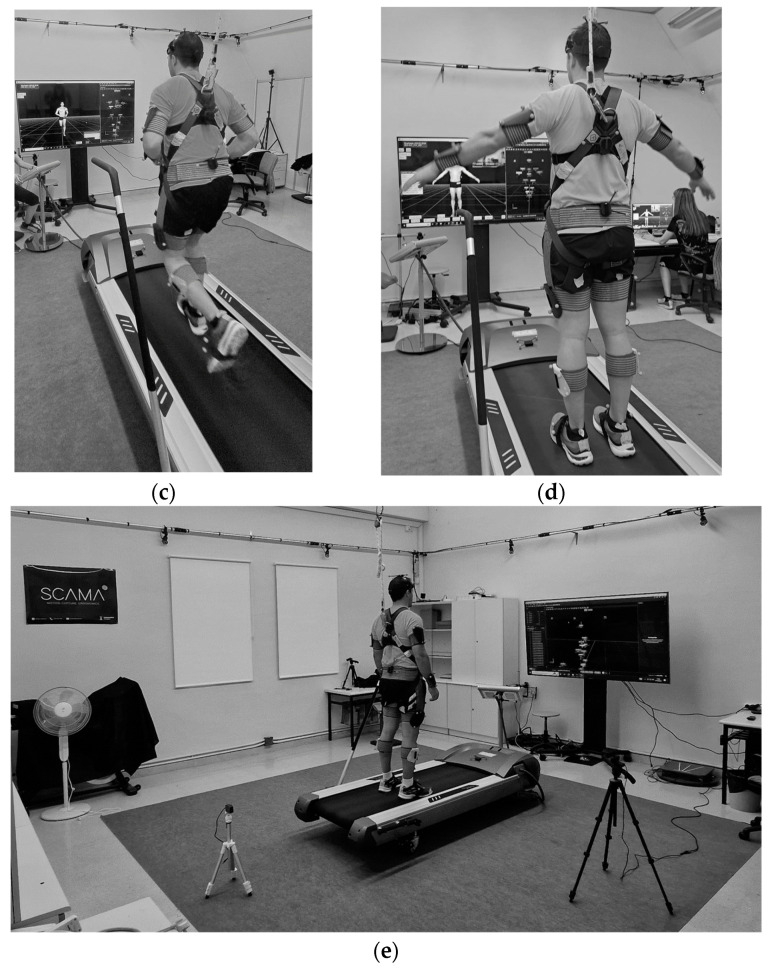
Equipment used in the KeepRunning test: (**a**) clusters of markers placed on the athlete; (**b**) clusters of markers; (**c**) athlete running on the treadmill; (**d**) calibration posture or Fitbody; (**e**) biomechanics laboratory.

The KeepRunning test is based on the MoCap Move Human Sensors (MH) optical system developed by the IDERGO Research Group. MH is described and evaluated by Marin et al. [20], and is based on 13 clusters of markers fixed on elastic bands placed on the studied body segments (Figure 1a) and a set of 12 infrared cameras to capture the position and orientation of the clusters of markers. The cameras are Opti-Track Flex 13 and are connected to Motive software (v. 2.3.2, NaturalPoint, Inc., P.O. Box 2317 Corvallis, OR 97339, USA) [39]. Optical technology is selected over inertial sensors due to its precision [20,22] in order to have the most accurate tool to prevent injuries in the KeepRunning test.

Each cluster of markers is a set of three passive reflective markers placed on a rigid 3D-printed surface (Figure 1b) and is unambiguously recognised by Motive software. The cameras work as optical sensors because they emit a beam of infrared light that is reflected by the passive reflective markers and captured by the camera lenses.

All data captured by the MoCap optical system were transferred in real time to the Motive software that captures the motion at 120 Hz, and streams it to a digital human model or avatar in Vizard software (v.7.4, WorldViz, Santa Barbara, CA, USA, 2020) while the subject is running in their usual running shoes on a treadmill (model EXE T800 modified with the control panel placed independently; Figure 1c). The avatar dimensions are adjusted to the runner’s anthropometry which is achieved by doing an anatomical calibration before capture known as Fitbody (Figure 1d) [20].

The MH software (v.102.06, University of Zaragoza, Zaragoza, Spain, 2023) based on Python 3.8 and the Pandas and Matplotlib libraries were integrated, which allowed for processing and analysis. In addition, these software programs export the data in an Excel-compatible format [40].

The MH system is configured to record live video with two webcams synchronised with the MH software: the back view of the athlete in the running direction and the side view of the treadmill (Figure 1e). These views have been chosen by the specialist physiotherapist because he claims that they provide useful information in the runner’s assessment.

#### 2.1.2. Data Provided by KeepRunning

The software programmes provide raw data of the runner’s movement. The aim is to select and provide variables to allow the clinician or coach to interpret the results from this data. Thus, running events that correspond to the characteristic instants in running must be defined because they are considered as a reference to determine the formulas that define the variables. In addition, these events allow the motion pattern analysis of the studied subject.

According to the literature, different terminology exists to define running events, which can be mainly divided into stance phase and swing phase. Thus, ground contact during running can be described as the stance phase [28,41,42], which is usually divided into three main phases: initial contact, midstance and propulsion [28] or toe off [42]. The swing phase [41,42,43,44] is characterised by two zones in which both feet are in the air, called float [41], flight phase [42] or double float [43,44], and another in which only the opposite foot is supported, defined as the swing [41] or stance of the opposite foot [42]. Table 1 summarizes this terminology and compares it to the events chosen in this research.

**Figure 2:**
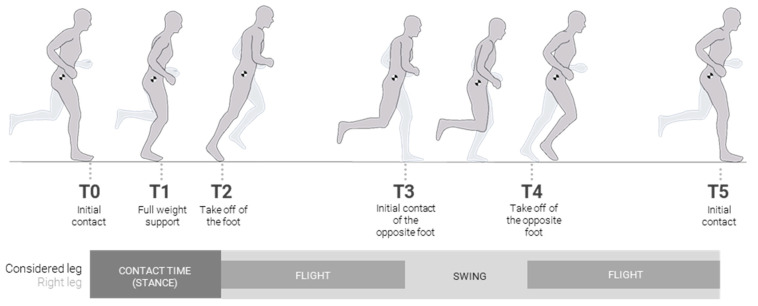
Running events in this research.

Based on the existing nomenclature, the events chosen for this research are illustrated in Figure 2, ranging from T0 to T5, corresponding to a complete stride, with the T5 event being the T0 instant of the following stride. These events were recorded for both the left and right leg strides.

The detection of the running events in each stride, corresponding to moments of foot contact with the ground, is usually performed using force plates placed on the laboratory floor or instrumented templates [20]. However, in this case, an algorithm described by Marin et al. [20] was applied in the MH software. The detection is based on the curve of the absolute displacement of the centre of the ankle joint on the Z-axis, and the same curve in the opposite foot. The MH system has rules for detecting running events (Table 2) based on searching and identifying the maximums and minimums of these curves without additional instrumentation.

Once the events were described, the variables most commonly used in the running analysis were selected based on the bibliographic review, complemented with the criteria from the research team which is composed of a physiotherapist and engineers specialising in biomedicine (e.g., in this research the clusters of markers on the arms have been used to obtain Dist.CoM-Hand because the physiotherapist considers it relevant to know if the runner moves the arms enough during running). Table 3 and Table 4 present the selected variables in this research and their descriptions, which are the result of the review and synthesis process, referring to the running events to allow a comparison between the measurement sessions or between subjects.

The (1) spatio-temporal, (2) kinetic, (3) stabilometric and (4) kinematic variables can be differentiated. The variables focus on the contact time or stance phase (T0 to T2) because it is the phase of running that seems to have the most influence on the occurrence of injuries [28,41]. However, events from T0 to T5 have been described in this research because some variables such as stride cadence, stride length or CoM vertical oscillation are calculated considering the complete stride (T0 to T5).

Kinematic variables are dimensions that depend on the movement of each body segment, allow the quantification of the movement ranges of each joint during the running cycle and are measured in degrees. Spatio-temporal variables depend on the movement of the whole body and quantify the distances and times of the running pattern. Kinetic variables consider the forces that cause the movement. Finally, stabilometric variables refer to the CoM.

### 2.2. Test–Retest Experiment

#### 2.2.1. Sample

A total of 32 people participated in this study to assess the reproducibility of the test, of which 16 were female and 16 were male (age: 30 ± 9.5 years; height: 170.1 ± 8.0 cm; weight: 67.6 ± 9.9 kg; body mass index: 23.3% ± 2.5%; foot length: 25.6 ± 1.4 cm; and abdominal perimeter: 78.4 ± 8.1 cm). The sample size was determined on the basis of other publications with similar characteristics to this research [13,14,15,16,17,18,19,20]. The inclusion criteria in this study were to (i) have a running training routine of at least 15 km per week, regardless of the level; (ii) be of legal age (between 18 and 60 years old); (iii) be in good health with no vascular, neurological or vestibular problems; (iv) not have any musculoskeletal disorders of the lower body joints or injuries on test day; (v) be able to achieve a running speed of between 9 and 12 km/h on a treadmill for approximately 5 min. Volunteers should know if they achieve this speed according to their training. This speed was selected to ensure that the walk-to-run transition speed has been exceeded [54,55], and ensure characteristic running results.

The study was approved by the Research Ethics Committee of the Community of Aragon (CEICA; 21 December 2022). Prior to the start of the tests, all participants signed the written informed consent form, agreeing to undergo the tests and declaring that they understood the objective of the study.

#### 2.2.2. Experimental Protocol

**Figure 3:**
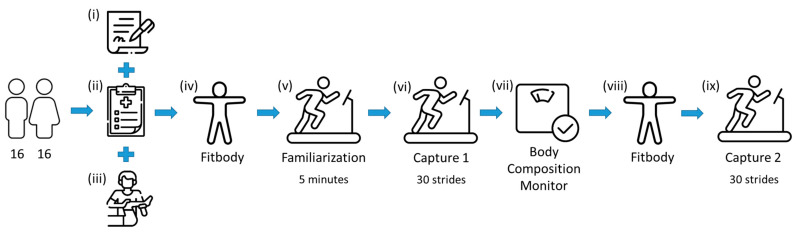
Test–retest experimental protocol (icons designed by Freepik, Iconriver and Md Tanvirul Haque [57]).

In relation to the evaluation protocol, an observational–descriptive–experimental test–retest study was conducted on a sample of 32 healthy subjects, consisting of repeating the running test twice with a homogeneous sample of participants under the same conditions. It was conducted on the same day with an interval of 20 min between the test and retest. In this interval, the clusters of markers were removed from the lower extremities. In addition, to characterise the participants, a weight scale (OMRON, Body Composition Monitor BF511) was used to measure the height, abdominal perimeter and foot length. Afterwards, the clusters of markers were relocated to account for the errors arising from the positioning of the markers on the body segments. The full test–retest protocol is presented in Figure 3 and consists of:

(i) Signing the informed consent;
(ii) Filling in an anamnesis sheet;
(iii) Manual examination by a physiotherapist specialised in the analysis and assessment of musculoskeletal injuries;
(iv) Anatomical calibration or Fitbody;
(v) 5 min treadmill adaptation time at a comfortable speed chosen by the subject (9.0 ± 0.9 km/h);
(vi) Capture of 30 complete running cycles;
(vii) Removal of the clusters of markers and characterisation of the participant using the body composition monitor and anthropometric measurements;
(viii) Anatomical calibration or Fitbody;
(ix) A second capture of 30 cycles at the same speed.

According to Kribus-Shmiel et al. [56], statistical stability and normality could be achieved with 23 strides or more, which is a sufficient number of cycles to represent average behaviour. In this work, we chose 30 cycles to ensure such stability.

Before the running is captured, the rater has to perform a Fitbody on the athlete and the runner is required to adapt to the treadmill for 5 min and observe that the pace is suitable for recording the movement. The total duration of the KeepRunning test is approximately 20 min.

Errors from testing are minimised by implementing a number of actions:

(i) The same rater coordinates the testing of all study participants.
(ii) Cluster of markers placement guidelines explained in Marin et al. [20] are followed.
(iii) The rater stands in front of the runner in the anatomical calibration imitating the posture to be adopted. This ensures that the runner adopts the correct calibration posture.

#### 2.2.3. Data Analysis

Once the variables were defined from the running events, reproducibility was obtained corresponding to the values of the 30 strides for each test (test and retest) of the 32 participants, characterised by the intraclass correlation coefficient (ICC) and MDC. The ICC is an index that varies between 0 and 1, determining the similarity between the test–retest results, where a score of 1 indicates that the test and retest measurements are the same [16]. Furthermore, MDC is defined as the amount of change that must occur in a variable to ensure that the change is not the result of measurement error, test process or protocol, instrumentation or random variation in a person’s movement [58,59]. Therefore, MDC represents the variability of the measurements of each variable. If a change in a variable below its MDC value is detected, it would not be considered relevant because it is less than the variability of the test [20].

ICC and MDC are extended indices in these types of studies. ICC is used because it is non-dimensional and allows measurement of the general concordance between two or more measurements that involve variables of a quantitative nature [60] according to standardised criteria [13,14,15,16,17,18,19]. MDC is used because it is applicable to the evaluation and comparison of a variable between two different captures.

The MDC values at 95% confidence were calculated using Equation (1) [14,16,58]:

(1) $$MDC95=1.96\;\sqrt{2}\;SEM;$$

(2) $$SEM={SD}_{pooled}\;\sqrt{1-ICC}\;$$

(3) $${SD}_{pooled}=\sqrt{\frac{\left(n_{1}-1\right){SD}_{1}^{2}+(n_{2}-1){SD}_{2}^{2}}{n_{1}+n_{2}-2}}$$

where SD denotes the *pooled* average of the standard deviation in the test and retest, ICC represents the intraclass correlation coefficient, SEM indicates the standard error of the measure, n_1_ is the sample size for group 1 and n_2_ for group 2. ICC was calculated from Python and Matplotlib libraries using the dataset from test and retest. Lower MDC values are better because they indicate higher reproducibility, and therefore more reliable results are obtained.

## 3. Results

**Figure 4:**
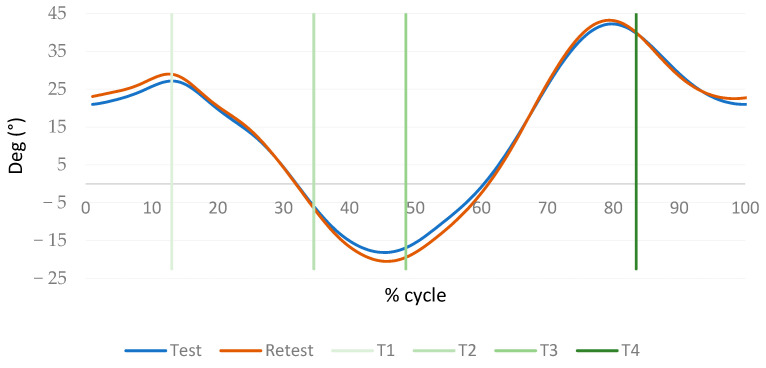
Example of the mean movement curve of the hip flexion–extension (Hip.FlexExt.) for the right leg.

The results of the test–retest reliability (ICC and MDC) come from the raw data corresponding to the 30 cycles measured at 120 Hz while performing the test and retest on each participant. This raw data corresponds to the movements of each body segment in the three anatomical planes. After detecting gait events and separating the movement of each body segment, movement curves where the mean values of the 30 cycles for each runner are calculated. An example of the mean movement curve obtained for hip flexion–extension (Hip.FlexExt.) for the right leg is shown in Figure 4. The vertical lines represent the events identified by the algorithm described, the blue line the test and the orange line the retest. These curves were used to calculate the reproducibility (ICC and MDC) of each variable in order to evaluate and validate the KeepRunning test, which is the main objective of this research.

**Figure 5:**
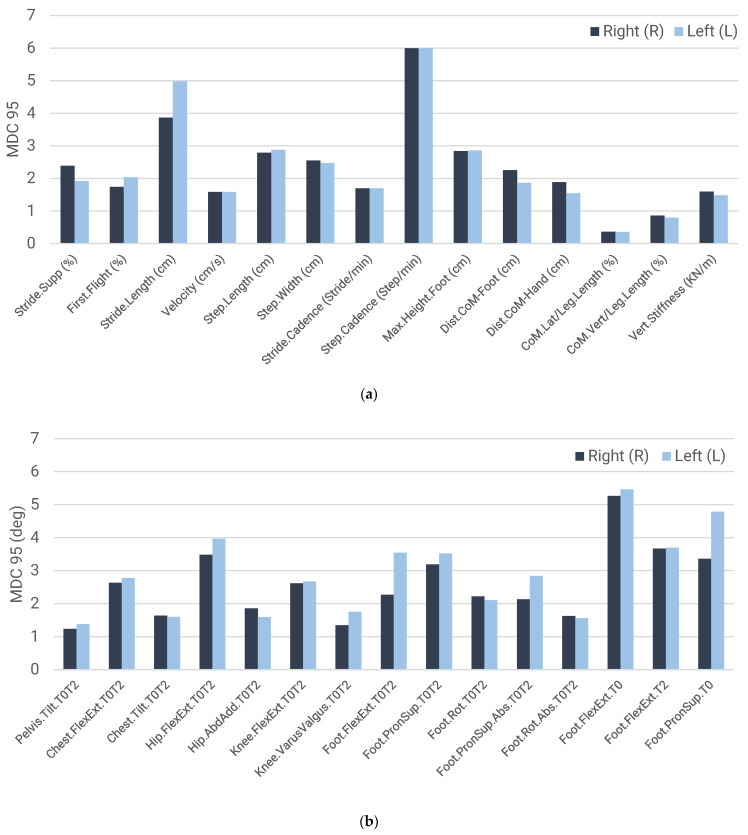
MDC 95 results for the right- and left-hand sides: (**a**) spatio-temporal, stabilometric and kinetic variables; (**b**) kinematic variables.

Table 5 and Table 6 and Figure 5 present the results of the test–retest reproducibility study using the optical MH system. The tables include the mean (µ) and standard deviation (SD) for the test and retest obtained for the right (R) and left (L) leg of all participants using the data recorded. In addition, the differences between test–retest means and the reproducibility, represented by the ICC and MDC 95 indices, are presented.

The last column contains the upper and lower limits which represent limits of normality for each variable (i.e., every value inside these limits will be considered inside the normality of the range provided by this study). These limits are the average obtained by adding and subtracting twice the SD from the mean for each variable (Equation (4)), ensuring the inclusion of 95% of the population. The mean value corresponds to the mean of the pre-post means, and the SD is the *pooled* average of the SDs (SD*pooled*).

(4) Limits=μ(prepost)±2·SD

Figure 5 graphically presents the MDC results comparing the values obtained in both legs to facilitate the interpretation of the results presented in the tables.

Table 5 and Table 6 show ICC values. They are close to 1 in most of the variables, which indicates good reproducibility (i.e., the results obtained in the test and retest are quite similar). The MDC values are between 0.36 and 6.00 for the set of spatio-temporal, kinetic and stabilometric variables, while the kinematic parameters are between 1.23° and 5.46°. These results must be compared with those obtained in other studies to verify the level of reproducibility. Good reproducibility would show the applicability of KeepRuning to aid coaches and clinicians to assess how runners modify their running technique between two successive captures.

## 4. Discussion

This study presents a running analysis test based on the optical MoCap on a treadmill called KeepRunning, aimed at injury prevention to allow coaches and clinicians to follow up with individual athletes objectively, following their indications. The running events and most representative and interpretable variables in the prevention of RRMIs were defined to design this test. Additionally, the reproducibility (ICC and MDC) of these parameters was calculated from test–retest reliability to evaluate the test.

Overall, this study provides knowledge in relation to the running events and the presented variables, providing a critical synthesis of the related literature. An evaluation framework for running events was established due to the considerable variability in the events used in the running analysis and their nomenclature [28,41,42,43]. Moreover, the selection of variables is one of the significant challenges of running analyses and MoCap, in general [26,27,28,29,30,31,32]. The presented contribution can simplify the analysis of other research and, above all, encourage the practical application of this technology to prevent RRMIs.

Thus, the discussion is structured into three sections. Section 4.1 discusses the quantitative results of the reproducibility of the KeepRunning test, obtained with the evaluation of 32 healthy runners. The reproducibility study allows the definition of the variability of each variable (i.e., the consistency of the variables), which includes different aspects: the variability of the instrument, the inherent variability of the person and their learning (test duration, visual condition or position of the feet) and the procedures and protocols applied to perform the test. Next, Section 4.2 details the applicability and usage considerations of the test, allowing the individualised analysis of runners following the indications of a coach comparing one measurement session with a subsequent session. Finally, Section 4.3 presents the limitations and future work of the study.

### 4.1. Discussion of the Test–Retest Results

In relation to the reproducibility results, the mean value of the ICC for all variables is 0.94 ± 0.05 (i.e., the results obtained in the test and retest are similar to each other because the ICC value is very close to unity), indicating good agreement between the two tests. Considering the existing ICC classifications, it is possible to appreciate that the ICC obtained in the present work is ‘excellent’ (0.90 ≤ ICC ≤ 1.00) [13,14,16,17] and ‘almost perfect’ (ICC ≥ 0.80) [18,19], indicating excellent agreement in the results. Additionally, the ICC is below 0.80 in only one case: Foot.PronSup.L T0 (ICC = 0.77, Table 6). These results demonstrate a high concordance between results.

To summarise the reproducibility data in terms of the MDC of the running, the variables were placed in different groups according to their units of measurement. The values in Table 7 are the mean and SD of these variable groups. A smaller value of MDC indicates that the variable has a higher reproducibility (i.e., the whole sample of people who completed the test followed a similar pattern of movement between the tests and retests).

To determine whether a good MDC result was obtained, we first refer to the study by Okahisa et al. [14], where they indicate that ‘a systematic review of the reliability of 3D motion analysis suggested that the limit of measurement error that is considered acceptable is less than 5°’. They also stated that ‘the reliability of an MDC of less than 3° rather than an MDC of 5° may be sufficient to detect clinically significant changes’. Thus, the average MDC of angular kinematic variables (2.73 ± 1.16 for running; Table 7) is close to and even slightly below this 3° to 5° range.

Table 8 and Table 9 list the MDC values from the literature and the present work. The following criteria were used for the selection of similar studies: experiments where the subjects (1) ran on the ground or a treadmill, (2) were healthy and (3) young, and (4) the studies provided the MDC value. Notes about the data selected in the various studies (Table 9) are necessary to provide comparable values.

From a general perspective, the mean MDCs in other studies were less reproducible (higher values) than those obtained in this work. Therefore, the reproducibility in the present work was better than the average found in the literature. Table 8 presents the values from this study that are better than those in the literature in bold. However, some variables in this work revealed higher MDC values than those found in other studies. Of these values, the only ones with a reasonable difference in magnitude were flexion–extension and pronation–supination of the foot at initial contact, which may be due to the different method of detecting the initial contact compared to the other studies.

Nevertheless, the results were compared with caution. We aimed to determine the maximum similarity to other studies using the mentioned criteria. However, these studies used different capture systems, parameters that were not precisely the same and, in some cases, different timing between testing and retesting. In addition, MDCs were obtained at different speeds depending on the study. These factors may explain the difference in the results. Additionally, by introducing considerably long delays between the testing and repetition, the instrumentation placement error, even if performed by the same rater, combined with the participant’s condition on different days, could influence the poorer results obtained in these studies.

In contrast, according to other studies [27,61], running on a treadmill is not the same as running on the ground. This difference is reflected in the values of kinematic and kinetic parameters. However, Table 8 reveals no noticeable difference between studies performed on the ground and those performed on the treadmill in terms of reproducibility.

### 4.2. Applicability and Usage Considerations of KeepRunning

Beyond the reproducibility results, which demonstrate that KeepRunning is a valid test to take successive measurements of a runner, it is interesting to describe how this test could be applied in daily clinical practice or to elaborate a future project training. In a scenario where KeepRunning is used for a runner who aims to improve his running technique, this runner would perform a running capture (pre-test) following the described protocol. With the gathered information and clinical observation, a professional could provide a series of technical recommendations for the runner to execute for a certain period, for example, 4 months, with the intention of modifying certain biomechanical parameters. Then, the runner would perform a new capture (post-test).

In this way, KeepRunning allows a comparison between captures. This comparison could make it easier for the professional to objectify how the running pattern has evolved between evaluations. Therefore, this system could check the influence of the recommendations, be more effective in establishing new indications, and more closely reach a biomechanical pattern that is less harmful to the runner with successive cycles of measurement, guideline and remeasurement.

However, it is necessary to determine how this comparison could be conducted at the level of data analysis between the results of the pre- and post-tests. For this purpose, we could objectively study the changes between the pre- and post-tests by applying a statistical test called magnitude-based decisions (MBD) [62], based on work by Batterham and Hopkins et al. [63,64], using MDC as the threshold of change, as did Furlan et al. [59]. MBD is not exempt from controversy; thus, some authors support [65,66,67] and others oppose [68,69] its application. However, we assumed this method to transmit a simple and interesting idea, considering a change relevant if it exceeds a specific threshold. This idea may not be applicable in all fields, but it makes sense in individual athlete monitoring [70]. Information about the MBD approach can be found in Excel spreadsheets, presentations, notes, and articles, all of which are available from sportsci.org [71].

To evaluate the statistical contrast for each runner and each variable, a pre-test data sample and twin post-test data sample were used, in which each data point corresponds to one running cycle. Thirty cycles can be measured for each runner to obtain this sample, as performed in this study, according to Kribus-Schmiel et al. [56] because it is sufficient to ensure statistical stability and normality. From these cycles, it is possible to obtain a sample of 30 values of each variable characterised by its corresponding mean and SD at the end of each capture.

The MBD method provides the probability that a change (defined by the confidence interval of the difference, CIdif) exceeds a specific threshold (−δ, +δ) selected by researchers in accordance with their objectives [70]. We propose such a comparison using an MBD approach to determine the confidence interval of the difference (between pre- and post-tests) in each variable in relation to the MDC threshold (+MDC, −MDC). The confidence interval of the difference is defined by the distribution of the difference of the samples for each variable between the pre- and post-tests. Thus, whether the interval of the difference in each variable between the pre- and post-tests is on the negative side, within, or on the positive side of the MDC threshold can be determined. MDC should be obtained through a test–retest to make this comparison, with the time between measurements equal to that of the clinical application, which is 4 months in this example.

If the interval of the difference is on the negative side, we obtain the percentage of negative differences (percentage of the interval outside the threshold), indicating the probability that the value of the variable in the post-test is significantly lower than in the pre-test. If the difference interval is within the threshold (or more than 95% is within the threshold), no significant difference exists, and the changes are trivial. If the difference interval is on the positive side, we obtain the percentage of positive differences (percentage of the interval outside the threshold), indicating the probability that the value of the variable in the post-test is significantly higher than in the pre-test [40]. Thus, the classification proposed by Hopkins et al. [64] can be used to assess the significance qualitatively, according to the percentage of negative, trivial or positive differences: <1%, most unlikely; 1% to 5%, very unlikely; 5% to 25%, unlikely; 25% to 75%, possibly; 75% to 95%, probably; 95% to 99%, very likely; and >99%, most likely).

**Figure 6:**
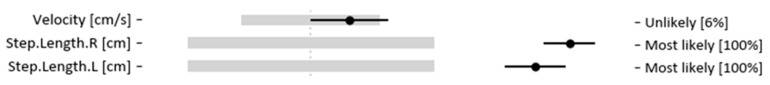
Extract from a graph of results of the statistical analysis.

**Figure 7:**
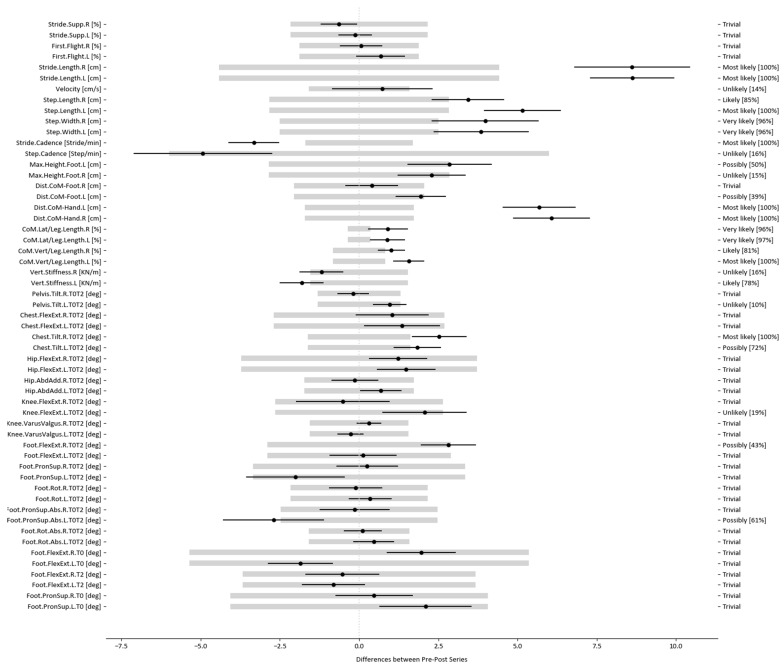
Graph of the full statistical analysis for all studied runner variables.

Figure 6 and Figure 7 graphically represent the results of this statistical analysis. The left area presents the analysed variables, and all or some of the variables presented in this study could be used. In the central area, the range of the difference is marked by a black line, characterised by a central point (the mean of the difference) and two extremes (the lower and upper 95% distribution limits). They also include the MDC of the variable, represented by light grey rectangles. Finally, the ranking of the change and its percentage significance are indicated on the right-hand side of the graph. Both figures display a fictitious example of the results graph of the statistical analysis.

The variables selected in this study from the literature and the research team’s criteria are presented in Figure 7. These variables correspond to the study of a specific runner. Figure 7 reveals the variables with a change between test and retest that overcomes the variability of the test. This change is ensured when the black line is completely outside the MDC threshold (grey rectangle) and is classified as most likely.

The clinician or coach needs to be able to interpret these changes and associate it with the influence it may have on the athlete’s running technique. Thus, the professional evaluates whether the change has been positive or negative based on his or her experience. In addition, the coach or clinician can check whether or not a proposed recommendation has been followed (e.g., if the coach considers that the runner needs to achieve a longer stride length and there is no change it means that the runner has not implemented the recommendation). If another athlete performs the test and retest, he or she could present significant changes in other different variables. This demonstrates the power of the graph obtained through KeepRunning because an individual analysis of each athlete can be conducted rapidly and easily interpreted.

The application of the KeepRunning test in daily clinical practice and the methodology of statistical results analysis could provide objective information to specialists responsible for the evaluation. The applied test could be helpful for runners who aim to modify their running technique or avoid RRMIs. Over time, this would build a database with the changes produced after establishing a set of technical recommendations by trainers or clinicians. General rules that associate these recommendations with specific changes in the different variables could be investigated with the aim of identifying recommendations leading to positive or negative changes in the running technique.

Thus, a professional could directly propose recommendations based on these rules to a runner who performed the test and met a specific biomechanical pattern corresponding to the existing database. This approach would facilitate and accelerate the work of the professional to provide an assessment of the runner and a proposal of recommendations to improve the running technique more efficiently.

### 4.3. Limitations and Future Work

The current work has several limitations. Due to the difficulty of recruiting volunteers and achieving a gender-equal sample, it was necessary to use occasional and experienced runners, but the two groups of runners have very different biomechanics and running techniques. Thus, this limitation can make it difficult to understand running mechanics and to obtain running patterns that can lead to RRMI.

The average age of all volunteer runners is relatively young because it was difficult to recruit older people. In addition, they were all healthy, non-injured athletes on the day of the test. These characteristics may not be representative of a general population. Therefore, another study should be conducted with a more diverse group of participants, considering age, fitness level and health status to generalise the results. Moreover, more specific or more restrictive studies could be conducted where only a group with certain characteristics is studied (e.g., a study of young experienced runners who have had plantar fasciitis). In this manner, risk factors for different injuries could be established.

The participants were fitted with a safety harness and were running on a treadmill; thus, they had difficulty achieving their natural running style. This condition has been able to generate higher MDCs in some variables and therefore provides a lower reproducibility or results in some values of the variables not being characteristic of the runner’s usual running technique. In the future, less cumbersome and simpler harnesses should be sought.

The literature review on running was complex. In particular, the study of MDC in running is not widespread. In many cases, studies do not describe the variables in detail or present graphs that represent them. This problem made it difficult to compare the data obtained with those in the literature.

In future work, the use of inertial outdoor sensors (e.g., on an athletics track) is proposed to make the conditions more realistic and more applicable to daily practice. Inertial technology is not limited to a laboratory environment like optical sensors but is designed to allow ambulatory measurements in the field simply and quickly [23,72]. KeepRunning could be used with inertial motion capture technology and other wearable technologies in the future. Inertial technology would save even more time in data collection than the current test but would lose accuracy. KeepRunning has been designed to be performed with inertial technology as well, so it would be necessary to compare results obtained with inertial and optical to see if the difference in precision is insignificant.

In addition, whether it is possible to compare running on a treadmill with running on a track is uncertain because a laboratory environment is not identical to the usual running environment and may result in different running kinematics and kinetics [73]. This comparison is made in other studies [74,75], where the authors claim differences exist in the results for various variables in both scenarios. In contrast, Pink et al. [76] affirmed that the results are similar for both cases. Therefore, more research is needed to establish whether there really are differences in the results of the variables that influence running when running on a treadmill or outdoors. The treadmill has been chosen in this study because it is the best way to conduct a study using optical motion capture, although this could negatively influence the biomechanics of the runner who is used to running on the track and alter his or her running technique.

A comparative study between running on a track and on a treadmill with inertial sensors could be conducted to see how the use of a treadmill really influences the variables. It is possible to apply KeepRunning because it has been designed to be done also with inertial sensors and it would only be necessary to compare the statistical graphs obtained in both tests to check if they are similar or not.

It would be of interest to apply this test periodically and regularly to runners to detect changes in running mechanics and follow up on their progression. This approach would make it easier to obtain transversal conclusions and common running patterns with positive or negative results in terms of sporting performance and prevention.

## 5. Conclusions

A powerful tool for running biomechanical analysis has been developed called KeepRunning. This test is aimed to provide objectivity in decision-making to clinicians and coaches. KeepRunning provides a rapid full body analysis of running technique, including 3D visualization in real-time combining spatio-temporal, stabilometric, kinetic and kinematic variables. This test favours the prevention of injuries because it facilitates the assessment of running evolution between two successive measurements, one before and the other after the training.

The variables defined in this study were the result of the combination between a literature review and the criteria of a multidisciplinary group formed by a physiotherapist specialized in biomechanics and engineers specialized in biomedical engineering. The presentation of the KeepRunning results has been designed in a simple format and easily interpretable manner by a clinician or a running coach.

In addition, KeepRunning’s reproducibility has been proved by a test–retest reliability test on 32 runners obtaining low values for MDC and ICCs close to one for each variable. Therefore, results obtained have demonstrated their consistency. In conclusion, KeepRunning can be considered a useful tool in daily practice that allows the clinician or coach a rapid running evaluation giving a practical application of optical MoCap technology that could facilitate work in preventing injury or improving sports performance.

## Figures and Tables

**Table 1:** Running events according to the literature and this study.

Phase	Literature	This Study	Description
Stance phase (T0–T2)	Initial contact [28]	Initial contact (T0)	Ground contact during running
	Midstance [28]	Full weight support (T1)	
	Propulsion [28] or toe off [42]	Take off of the foot (T2)	
Swing phase (T3–T5)	Float [41], flight phase [42] or double float [43,44]	Flight (T2–T3)	Both feet are in the air
	Swing [41] or stance of the opposite foot [42]	Swing (T3–T4)	Only the opposite foot is supported
	Float [41], flight phase [42] or double float [43,44]	Flight (T4–T5)	Both feet are in the air

**Table 2:** Criteria established for event identification.

Events	Running Cycle
T0	Initial contact of the foot with the heel, midfoot or toe (start of the running cycle of that foot)
T1	Supporting the full weight on the considered foot, which is aligned with the CoM
T2	Take off of the foot under consideration (start of the first flight with both feet)
T3	Opposite foot contact with the heel, midfoot or toe (start of the opposite foot strike and end of the first flight)
T4	Take off of the opposite foot (start of the second flight)
T5 = T0	Again, initial foot contact (100% of the cycle is completed)

**Table 3:** Spatio-temporal, kinetic and stabilometric variables.

Category	Name	Description	References
Spatio-temporal	Stride.Supp (%)	Percentage of support along the stride (from T0 to T2)	[28,31,45]
	First.Flight (%)	Percentage of flight (both feet) between T2 and T3 with respect to the full stride	Defined by us
	Stride.Length (cm)	Length of a full stride (T0 to T5)	[26,27,30,44,45,46,47,48]
	Velocity (cm/s)	Average velocity throughout the stride	Defined by us
	Step.Length (cm)	Distance between the feet in the sagittal plane between T0 and T3	[28,29,31,44,49,50]
	Step.Width (cm)	Distance between the feet in the frontal plane between T0 and T3	Defined by us
	Stride.Cadence (Stride/min)	Number of strides completed in 1 min	[26,27,30,48]
	Step.Cadence (Step/min)	Number of steps completed in 1 min	[26,28,29,31,45,50]
	Max.Height.Foot (cm)	Maximum height reached by the foot opposite to the foot under study during the stance phase (T0 to T2)	Defined by us
Stabilometric	Dist.CoM-Foot (cm)	Sagittal plane distance between the subject’s centre of mass and malleolus	[47]
	Dist.CoM-Hand (cm)	Distance in the sagittal plane between the subject’s centre of mass and wrist	Defined by us
	CoM.Lat/Leg.Length (%)	Ratio between the lateralisation of the subject’s centre of mass and leg length	Defined by us
	CoM.Vert/Leg.Length (%)	Ratio between the vertical oscillation of the subject’s centre of mass and leg length	[30,47]
Kinetic	Vert.Stiffness (KN/m)	Vertical stiffness of the subject in running according to the spring-mass model	[18,28,49,51]

**Table 4:** Kinematic variables.

Name	Description	References
Pelvis.Tilt.T0T2 (deg)	Lateralisation of the pelvis. Maximum lateral tilt with respect to T0 during the stance phase (from T0 to T2)	[13,16,30,47,52]
Chest.FlexExt.T0T2 (deg)	Dorsal flexion–extension. Average anteroposterior tilt during the stance phase (from T0 to T2)	[16,19,30,47,50]
Chest.Tilt.T0T2 (deg)	Dorsal lateralisation. Maximum lateral tilt with respect to T0 during the stance phase (from T0 to T2)	[13,16]
Hip.FlexExt.T0T2 (deg)	Hip flexion–extension. Angle described during the stance phase (T0 to T2)	[14,16,26,30,52,53]
Hip.AbdAdd.T0T2 (deg)	Hip abduction–adduction. Angle described from T0 to the maximum during the stance phase (T0 to T2)	[13,14,16,26]
Knee.FlexExt.T0T2 (deg)	Knee flexion–extension. Angle described from T0 to the maximum during T0 and T2	[14,16,19,26,30,47,53]
Knee.VarusValgus.T0T2 (deg)	Knee varus–valgus. Angle described during the stance phase (from T0 to T2)	[14,16]
Foot.FlexExt.T0T2 (deg)	Flexo-extension of the foot. Angle described from T0 to the maximum during the stance phase (from T0 to T2) with respect to the tibia	[14,16,17,53]
Foot.PronSup.T0T2 (deg)	Pronation–supination of the foot. Angle described from T0 to the maximum during the stance phase (T0 to T2) with respect to the tibia	[16,17,27,47]
Foot.Rot.T0T2 (deg)	Foot internal–external rotation. Angle described during the stance phase (from T0 to T2) with respect to the tibia	[14,16,17]
Foot.PronSup.Abs.T0T2 (deg)	Pronation–supination of the foot. Angle described from T0 to the maximum during the stance phase (from T0 to T2) with respect to the ground	[17]
Foot.Rot.Abs.T0T2 (deg)	Foot internal–external rotation. Angle described during the stance phase (from T0 to T2) with respect to the ground	[17]
Foot.FlexExt.T0 (deg)	Flexo-extension of the foot. Angle at T0 with respect to the ground	[13,15,17,19,30,47,53]
Foot.FlexExt.T2 (deg)	Flexo-extension of the foot. Angle at T2 with respect to the ground	Defined by us
Foot.PronSup.T0 (deg)	Pronation–supination of the foot. Angle at T0 with respect to the ground	[17,19]

**Table 5:** Results of the test–retest for the spatio-temporal, stabilometric and kinetic variables.

	Variables		Test μ (SD)	Retest μ (SD)	Dif. μ (SD)	ICC	MDC 95	Limits
Spatio-temporal	Stride.Supp (%)	R	34.3 (3.9)	34.3 (3.6)	0.0 (1.7)	0.95	2.39	(26.9–42.0)
		L	34.6 (4.0)	34.5 (3.6)	−0.1 (1.4)	0.97	1.92	
	First.Flight (%)	R	15.6 (4.0)	15.4 (3.7)	−0.2 (1.2)	0.97	1.74	(8.1–23.1)
		L	15.5 (3.8)	15.8 (3.5)	0.3 (1.4)	0.96	2.04	
	Stride.Length (cm)	R	163.6 (19.6)	162.9 (19.2)	−0.8 (2.8)	0.99	3.87	(124.5–201.9)
		L	163.6 (19.7)	162.6 (18.9)	−1.1 (3.6)	0.99	4.97	
	Velocity (cm/s)		228.6 (23.9)	228.3 (24.2)	−0.2 (1.1)	1.00	1.59	(180.4–276.5)
	Step.Length (cm)	R	81.7 (10.3)	81.1 (9.8)	−0.6 (2.0)	0.99	2.79	(62.0–101.2)
		L	81.9 (9.5)	81.8 (9.6)	−0.2 (2.1)	0.99	2.88	
	Step.Width (cm)	R	8.2 (2.4)	8.6 (2.5)	0.4 (1.7)	0.86	2.55	(3.5–13.3)
		L	8.2 (2.4)	8.6 (2.5)	0.4 (1.7)	0.86	2.47	
	Stride.Cadence (Stride/min)		80.0 (4.2)	80.2 (4.0)	0.2 (1.2)	0.98	1.70	(72.0–88.2)
	Step.Cadence (Step/min)		160.4 (9.2)	161.4 (8.8)	0.9 (4.2)	0.94	6.00	(142.9–178.9)
	Max.Height.Foot (cm)	L	37.4 (8.0)	37.7 (7.9)	0.4 (2.0)	0.98	2.86	(20.9–55.3)
		R	38.6 (9.0)	38.8 (9.5)	0.2 (2.0)	0.99	2.84	
Stabilometric	Dist.CoM-Foot (cm)	R	14.7 (3.3)	14.6 (3.1)	−0.1 (1.6)	0.93	2.26	(8.2–21.2)
		L	14.8 (3.3)	14.6 (3.3)	−0.2 (1.3)	0.96	1.86	
	Dist.CoM-Hand (cm)	L	19.5 (4.1)	19.6 (3.9)	0.0 (1.1)	0.98	1.54	(11.5–28.1)
		R	20.0 (4.3)	20.2 (4.3)	0.2 (1.3)	0.97	1.89	
	CoM.Lat/Leg.Length (%)	R	1.6 (0.4)	1.6 (0.5)	0.0 (0.3)	0.93	0.36	(0.7–2.6)
		L	1.7 (0.5)	1.7 (0.5)	0.0 (0.2)	0.93	0.36	
	CoM.Vert/Leg.Length (%)	R	10.4 (1.6)	10.6 (1.6)	0.1 (0.6)	0.96	0.86	(7.3–13.4)
		L	10.2 (1.5)	10.3 (1.3)	0.1 (0.6)	0.96	0.79	
Kinetic	Vert.Stiffness (KN/m)	R	18.3 (2.7)	18.0 (2.6)	−0.3 (1.1)	0.95	1.60	(13.0–23.7)
		L	18.6 (2.7)	18.6 (2.6)	−0.1 (1.1)	0.96	1.49	

µ: Average; SD: Standard deviation; Dif.: Mean difference between a subject’s tests; ICC: Intraclass correlation coefficient; MDC 95: Minimal detectable change at 95%; R: Right-hand side; L: Left-hand side.

**Table 6:** Results of the test–retest for the kinematic variables.

Variables		Test μ (SD)	Retest μ (SD)	Dif. μ (SD)	ICC	MDC 95	Limits
Pelvis.Tilt.T0T2 (deg)	R	6.1 (2.2)	5.9 (2.1)	−0.1 (0.9)	0.96	1.23	(1.9–10.2)
	L	6.2 (2.0)	5.9 (2.1)	−0.3 (1.0)	0.94	1.38	
Chest.FlexExt.T0T2 (deg)	R	10.3 (3.8)	10.4 (3.5)	0.1 (1.8)	0.93	2.63	(2.7–17.9)
	L	10.1 (4.2)	10.4 (3.7)	0.3 (1.9)	0.94	2.78	
Chest.Tilt.T0T2 (deg)	R	6.7 (1.8)	6.7 (1.9)	0.0 (1.1)	0.90	1.64	(3.2–10.9)
	L	7.5 (1.9)	7.3 (2.2)	−0.2 (1.1)	0.92	1.60	
Hip.FlexExt.T0T2 (deg)	R	33.1 (4.8)	32.9 (4.0)	−0.2 (2.4)	0.92	3.48	(24.6–42.3)
	L	34.0 (4.6)	33.6 (4.3)	−0.4 (2.7)	0.90	3.97	
Hip.AbdAdd.T0T2 (deg)	R	8.5 (3.2)	8.3 (3.2)	−0.2 (1.3)	0.96	1.86	(1.6–14.8)
	L	8.0 (3.5)	7.9 (3.4)	−0.1 (1.1)	0.97	1.59	
Knee.FlexExt.T0T2 (deg)	R	27.5 (5.9)	26.9 (5.8)	−0.6 (1.9)	0.97	2.62	(15.7–38.7)
	L	27.4 (5.6)	27.0 (5.7)	−0.4 (1.9)	0.97	2.67	
Knee.VarusValgus.T0T2 (deg)	R	4.9 (1.9)	5.0 (2.0)	0.1 (0.9)	0.94	1.35	(1.4–8.3)
	L	4.8 (1.4)	4.7 (1.6)	0.0 (1.2)	0.83	1.75	
Foot.FlexExt.T0T2 (deg)	R	17.2 (4.0)	17.4 (3.8)	0.2 (1.6)	0.96	2.27	(8.4–25.8)
	L	17.1 (4.8)	16.7 (4.7)	−0.4 (2.5)	0.93	3.54	
Foot.PronSup.T0T2 (deg)	R	15.9 (3.3)	15.7 (3.2)	−0.2 (2.2)	0.88	3.19	(8.9–23.5)
	L	16.3 (3.8)	17.0 (4.2)	0.7 (2.4)	0.90	3.52	
Foot.Rot.T0T2 (deg)	R	11.6 (2.7)	12.0 (3.1)	0.4 (1.5)	0.93	2.22	(5.3–17.8)
	L	10.9 (3.2)	11.7 (3.4)	0.8 (1.5)	0.95	2.11	
Foot.PronSup.Abs.T0T2 (deg)	R	12.9 (2.8)	12.8 (2.8)	−0.1 (1.5)	0.92	2.13	(6.6–18.6)
	L	12.2 (3.1)	12.6 (3.3)	0.4 (2.0)	0.90	2.84	
Foot.Rot.Abs.T0T2 (deg)	R	6.7 (2.1)	6.6 (2.0)	−0.1 (1.1)	0.92	1.63	(2.5–10.7)
	L	6.6 (2.1)	6.5 (2.0)	−0.1 (1.1)	0.92	1.56	
Foot.FlexExt.T0 (deg)	R	−11.2 (9.9)	−11.6 (9.4)	−0.4 (3.7)	0.96	5.26	(−31.9–8.8)
	L	−11.6 (11.0)	−11.7 (10.4)	−0.1 (3.9)	0.97	5.46	
Foot.FlexExt.T2 (deg)	R	44.9 (4.2)	45.2 (3.9)	0.4 (2.5)	0.89	3.67	(36.6–52.9)
	L	44.4 (4.3)	44.5 (4.0)	0.2 (2.5)	0.90	3.70	
Foot.PronSup.T0 (deg)	R	−12.4 (3.0)	−12.7 (2.5)	−0.3 (2.2)	0.81	3.36	(−19.1–−6.4)
	L	−12.8 (3.5)	−13.0 (3.7)	−0.2 (3.1)	0.77	4.78	

µ: Average; SD: Standard deviation; Dif.: Mean difference between a subject’s tests; ICC: Intraclass correlation coefficient; MDC 95: Minimal detectable change at 95%; R: Right-hand side; L: Left-hand side.

**Table 7:** Minimal detectable change (MDC) results from the KeepRunning test.

	MDC Running
	Average ± SD
Variables related to the detection of support in (%) Stride.Supp; First.Flight.	2.82 ± 1.30
Step-related variables in (cm) Stride.Length; Step.Length; Step.Width; Max.Height.Foot.	3.15 ± 0.85
CoM distances in (cm) Dist.CoM-Foot; Dist.CoM-Hand.	1.89 ± 0.29
CoM related variables in (%) CoM.Lat/Leg.Length; CoM.Vert/Leg.Length.	0.59 ± 0.27
Vertical stiffness variable in (KN/m) Vert.Stiffness.	1.54 ± 0.08
Velocity variable in (cm/s) Velocity.	1.59
Stride cadence variable in (Stride/min) Stride.Cadence.	1.70
Step cadence variable in (Step/min) Step.Cadence.	6.00
Angular kinematic variables (°) Joint ranges of motion.	2.73 ± 1.16

Notes: In all cases, the average was taken of the variables, including right and left legs.

**Table 8:** Comparison of MDC results of the selected variables with those of other studies.

		MDCs in the Literature									MDCs in This Study	
		[14]		[13]		[15]	[16]	[17]	[18]	[19]	**MH**	
**Spatio-temporal variables**		**PiG**	**CGM2**	**Dr**	**Iz**						**Dr**	**Iz**
Stride.Supp (%)		-	-	-	-	1.8	-	-	-	-	2.4	1.9
Stride.Length (cm)		-	-	-	-	5.1	-	-	-	12.4	**3.9**	**5.0**
Step.Length (cm)		-	-	-	-	2.8	-	-	4.7	6.2	**2.8**	**2.9**
Step.Width (cm)		-	-	-	-	1.4	-	-	-	1.5	2.5	2.5
Step.Cadence (Step/min)		-	-	-	-	4.5	-	-	7.0	11.0	**6.0**	**6.0**
CoM.Vert/Leg.Length (%)		-	-	-	-	-	-	-	-	1.5	**0.9**	**0.8**
Vert.Stiffness (KN/m)		-	-	-	-	-	-	-	2.7	-	**1.6**	**1.5**
		[14]		[13]		[15]	[16]	[17]	[18]	[19]	**MH**	
**Kinematic variables (°)**		**PiG**	**CGM2**	**Dr**	**Iz**						**Dr**	**Iz**
Pelvis.Tilt.T0T2		-	-	2.7	2.8	-	1.6	-	-	-	**1.2**	**1.4**
Chest.FlexExt.T0T2		-	-	-	-	-	10.4	-	-	2.2	**2.6**	**2.8**
Chest.Tilt.T0T2		-	-	1.8	1.8	-	1.7	-	-	-	**1.6**	**1.6**
Hip.FlexExt.T0T2		6.8	4.4	-	-	-	8.3	-	-	-	**3.5**	**4.0**
Hip.AbdAdd.T0T2		5.0	3.0	2.8	2.3	-	2.6	-	-	-	**1.9**	**1.6**
Knee.FlexExt.T0T2		5.2	3.4	2.9 (T1) *	3.8 (T1) *	-	6.0	-	-	6.1	**2.6**	**2.7**
Knee.VarusValgus.T0T2		4.2	1.8	-	-	-	4.8	-	-	-	**1.3**	**1.8**
Foot.FlexExt.T0T2		3.5	1.8	2.2 (T1) *	4.8 (T1) *	-	7.5	1.9	-	-	**2.3**	**3.5**
Foot.PronSup.T0T2		-	-	-	-	-	3.3	2.1	-	-	3.2	3.5
Foot.Rot.T0T2		13.9	6.4	-	-	-	3.1	1.8	-	-	**2.2**	**2.1**
Foot.PronSup.Abs.T0T2		-	-	-	-	-	-	1.7	-	-	2.1	2.8
Foot.Rot.Abs.T0T2		-	-	-	-	-	-	1.3	-	-	1.6	1.6
Foot.FlexExt.T0		3.5 *	1.8 *	2.0	2.3	1.7	6.9 *	1.5	-	4.3	5.3	5.5
Foot.PronSup.T0		-	-	-	-	-	7.2 *	2.4	-	1.2	3.4	4.8
Notes	[14]	* They do not provide ranges of movement. MDC from flexion peak during the stance phase was selected, except for Foot.FlexExt.T0. It was MDC in initial contact. We assume relative angles.										
	[13]	* They do not provide ranges of movement in the knee and foot. We took the MDC of the peaks in T1.										
	[16]	The * indicates relative angles, but we use absolute angles in Foot.FlexExt.T0, Foot.FlexExt.T2 and Foot.PronSup.T0.										

Values in **bold** have better MDCs than the averages in other studies.

**Table 9:** Details of the studies with which the present work has been compared.

MDCs		In the Literature							In This Study
Study details		[14]	[13]	[15]	[16]	[17]	[18]	[19]	MH
Experimental conditions		Floor	Treadmill	Treadmill	Treadmill	Floor	Treadmill	Treadmill	Treadmill
MoCap technology		Full body optical	Trunk, pelvis, lower body optical	Instrumented Treadmill	Trunk, pelvis, lower body optical	Trunk, pelvis, lower body optical	Instrumented Treadmill	Two external optical sensors	Full body optical
Sample		23 (7 F, 16 M)	21 (12 F, 9 M)	33 (17 F, 16 M)	16 (10 F, 6 M)	18 (18 M)	31 (13 F, 18 M)	24 (8 F, 16 M)	32 (16 F, 16 M)
Age		24.3 ± 4.2	28.1 ± 8.3	31.6 ± 7.4	34.4 ± 10.2	28 ± 7	34.42 ± 9.26	22.7 ± 2.6	30 ± 9.5
Rater		Marker placements—Same	Rater 1–10 participants Rater 2–11 participants	Not found	Marker placements—Same	Marker placements—Same	Not found	Not found	Same
Time		5 days	One week	One week 30 min same day	Two weeks	At least 5 days	Same day with no time One week	5 min	20 min
Velocity (km/h)		9.0	10.0	9.0	11.5	13.3	12.0	10.0	9.0
Notes	[14]	PiG: Plug-in gait model; CGM2: Conventional gait Model 2.							
	[15]	Only data from the right side were further analysed. We compare them with the MDC within-day results.							
	[17]	It is unclear whether the data obtained are from the right and left sides averaged or only the right side.							
	[18]	They do not say what side was analysed. We compare this with the MDC within-day results.							
	[19]	Only the right leg of the participants was analysed.							

F: Female; M: Male.

## Data Availability

Data are contained within the article.
